# Ethyl 2-hydr­oxy-5,11-dioxopyrrolo[2,1-*c*][1,4]benzodiazepine-10-acetate

**DOI:** 10.1107/S1600536810006914

**Published:** 2010-03-03

**Authors:** S. Ourahou, M. Chammache, H. Zouihri, El Mokhtar Essassi, Seik Weng Ng

**Affiliations:** a Laboratoire de Chimie Organique Hétérocyclique, Pôle de Compétences Pharmacochimie, Université Mohammed V-Agdal, BP 1014 Avenue Ibn Batout, Rabat, Morocco; b CNRST, Division of UATRS Angle Allal Fassi/FAR, BP 8027 Hay Riad, 10000 Rabat, Morocco; c Department of Chemistry, University of Malaya, 50603 Kuala Lumpur, Malaysia

## Abstract

The title compound, C_16_H_18_N_2_O_5_, consists of a benzodiazepinedione system fused to a pyrrole system. The seven-membered ring adopts a boat-shaped conformation (with the methine C atom as the prow); the five-membered ring adopts an enveloped-shaped conformation (with the hydr­oxy-bearing C atom as the flap). The hydr­oxy group is hydrogen bonded to the carbonyl O atom of an adjacent mol­ecule generating a zigzag chain in the crystal structure.

## Related literature

Pyrrolo[2,1-*c*][1,4]benzodiazepines are potent anti­biotics produced by *Streptomyces *species; see: Cargill *et al.* (1974 ▶). For the design of DNA inter-strand cross-linking as well as of conjugate agents to enhance the sequence selectivity and selectivity for tumor cells, see: Gregson *et al.* (2004 ▶).
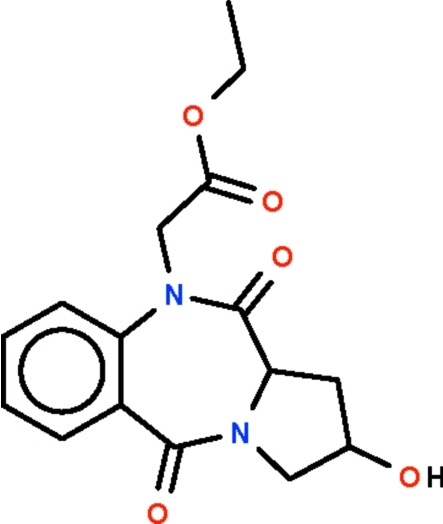


## Experimental

### 

#### Crystal data

C_16_H_18_N_2_O_5_
*M* *_r_* = 318.32
Orthorhombic, 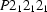

*a* = 8.5503 (2) Å
*b* = 9.6580 (2) Å
*c* = 18.2734 (4) Å
*V* = 1509.00 (6) Å^3^
*Z* = 4
Mo *K*α radiation
μ = 0.11 mm^−1^
*T* = 293 K
0.3 × 0.3 × 0.3 mm

#### Data collection

Bruker APEXII diffractometer
11809 measured reflections
1907 independent reflections
1765 reflections with *I* > 2σ(*I*)
*R*_int_ = 0.024

#### Refinement

*R*[*F* ^2^ > 2σ(*F* ^2^)] = 0.032
*wR*(*F* ^2^) = 0.098
*S* = 1.04
1907 reflections
213 parameters
1 restraint
H atoms treated by a mixture of independent and constrained refinement
Δρ_max_ = 0.21 e Å^−3^
Δρ_min_ = −0.17 e Å^−3^

### 

Data collection: *APEX2* (Bruker, 2005 ▶); cell refinement: *SAINT* (Bruker, 2005 ▶); data reduction: *SAINT*; program(s) used to solve structure: *SHELXS97* (Sheldrick, 2008 ▶); program(s) used to refine structure: *SHELXL97* (Sheldrick, 2008 ▶); molecular graphics: *X-SEED* (Barbour, 2001 ▶); software used to prepare material for publication: *publCIF* (Westrip, 2010 ▶).

## Supplementary Material

Crystal structure: contains datablocks global, I. DOI: 10.1107/S1600536810006914/bt5201sup1.cif

Structure factors: contains datablocks I. DOI: 10.1107/S1600536810006914/bt5201Isup2.hkl

Additional supplementary materials:  crystallographic information; 3D view; checkCIF report

## Figures and Tables

**Table 1:** Hydrogen-bond geometry (Å, °)

*D*—H⋯*A*	*D*—H	H⋯*A*	*D*⋯*A*	*D*—H⋯*A*
O3—H3⋯O1^i^	0.84 (1)	2.02 (2)	2.810 (2)	157 (4)

Symmetry code: (i) 
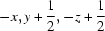
.

**Fig. 1.**
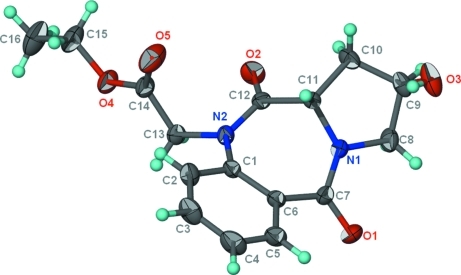
Anisotropic displacement ellipsoid plot (Barbour, 2001) of C16H18N2O5 at the 50% probability level; hydrogen atoms are drawn as arbitrary radius.

## supplementary crystallographic information

### Experimental

2-Hydroxy- pyrrolo[2,1-*c*][1,4]benzodiazepine-5,11-dione (0.5 g, 2.15 mmol), ethyl bromoacetate (0.45 ml, 4.3 mmol), potassium carbonate (0.6 g, 4.3 mmol) along with a catalytic amount of tetra-*n*-butyl ammonium bromide were stirred in *N*,*N*-dimethylformamide (20 ml) for 24 h. After the completion of the reaction (monitored by TLC), the solid material was removed by filtration and the solvent evaporated under vacuum. Dichloromethane (20 ml) was added and the solution filtered. The solvent was removed and the product purified by recrystallization from ethanol to afford colorless crystals in 80% yield.

### Refinement

Carbon-bound H-atoms were placed in calculated positions (C—H 0.93-0.98 Å) and were included in the refinement in the riding model approximation, with *U*(H) set to 1.2–1.5*U*(C). The oxygen-bound H-atom was located in a difference Fourier map, and was refined isotropically with a distance restraint of O–H = 0.84±0.01 Å. Due to the absence of anomalous scatterers Friedel pairs were merged and the absolute configuration was arbitrarily set.

### Crystal data

**Table:**

C_16_H_18_N_2_O_5_	*F*(000) = 672
*M**_r_* = 318.32	*D*_x_ = 1.401 Mg m^−^^3^
Orthorhombic, *P*2_1_2_1_2_1_	Mo *K*α radiation, λ = 0.71073 Å
Hall symbol: P 2ac 2ab	Cell parameters from 6127 reflections
*a* = 8.5503 (2) Å	θ = 2.2–32.7°
*b* = 9.6580 (2) Å	µ = 0.11 mm^−^^1^
*c* = 18.2734 (4) Å	*T* = 293 K
*V* = 1509.00 (6) Å^3^	Block, colorless
*Z* = 4	0.3 × 0.3 × 0.3 mm

### Data collection

**Table:**

Bruker APEXII diffractometer	1765 reflections with *I* > 2σ(*I*)
Radiation source: fine-focus sealed tube	*R*_int_ = 0.024
graphite	θ_max_ = 27.5°, θ_min_ = 3.1°
φ and ω scans	*h* = −10→9
11809 measured reflections	*k* = −12→12
1907 independent reflections	*l* = −23→23

### Refinement

**Table:**

Refinement on *F*^2^	Primary atom site location: structure-invariant direct methods
Least-squares matrix: full	Secondary atom site location: difference Fourier map
*R*[*F*^2^ > 2σ(*F*^2^)] = 0.032	Hydrogen site location: inferred from neighbouring sites
*wR*(*F*^2^) = 0.098	H atoms treated by a mixture of independent and constrained refinement
*S* = 1.04	*w* = 1/[σ^2^(*F*_o_^2^) + (0.0709*P*)^2^ + 0.1392*P*] where *P* = (*F*_o_^2^ + 2*F*_c_^2^)/3
1907 reflections	(Δ/σ)_max_ = 0.001
213 parameters	Δρ_max_ = 0.21 e Å^−^^3^
1 restraint	Δρ_min_ = −0.17 e Å^−^^3^

### Special details

**Table:**

Geometry. All esds (except the esd in the dihedral angle between two l.s. planes) are estimated using the full covariance matrix. The cell esds are taken into account individually in the estimation of esds in distances, angles and torsion angles; correlations between esds in cell parameters are only used when they are defined by crystal symmetry. An approximate (isotropic) treatment of cell esds is used for estimating esds involving l.s. planes.

### Fractional atomic coordinates and isotropic or equivalent isotropic displacement parameters (Å^2^)

**Table:**

	*x*	*y*	*z*	*U*_iso_*/*U*_eq_	
O1	−0.0674 (2)	1.16444 (16)	−0.01855 (9)	0.0502 (4)	
O2	0.1577 (2)	1.04456 (15)	0.23744 (7)	0.0480 (4)	
O3	0.2180 (2)	1.50177 (16)	0.09188 (10)	0.0577 (5)	
H3	0.277 (3)	1.452 (3)	0.0664 (16)	0.077 (10)*	
O4	0.25606 (19)	0.66847 (14)	0.26892 (8)	0.0426 (4)	
O5	0.4250 (2)	0.8107 (2)	0.21372 (11)	0.0677 (6)	
N1	0.0805 (2)	1.20584 (14)	0.08027 (8)	0.0306 (4)	
N2	0.1825 (2)	0.92800 (14)	0.13177 (7)	0.0299 (3)	
C1	0.2136 (2)	0.92042 (17)	0.05498 (9)	0.0275 (4)	
C2	0.3094 (3)	0.81259 (19)	0.03067 (10)	0.0386 (4)	
H2	0.3605	0.7567	0.0645	0.046*	
C3	0.3287 (3)	0.7885 (2)	−0.04302 (12)	0.0478 (6)	
H3A	0.3932	0.7168	−0.0586	0.057*	
C4	0.2530 (3)	0.8699 (2)	−0.09410 (11)	0.0495 (6)	
H4	0.2638	0.8517	−0.1438	0.059*	
C5	0.1614 (3)	0.9784 (2)	−0.07053 (9)	0.0395 (5)	
H5	0.1116	1.0338	−0.1050	0.047*	
C6	0.1411 (2)	1.00772 (17)	0.00397 (8)	0.0289 (4)	
C7	0.0428 (2)	1.13048 (18)	0.02157 (9)	0.0325 (4)	
C8	0.0024 (3)	1.33736 (19)	0.09788 (12)	0.0403 (4)	
H8A	−0.0890	1.3219	0.1282	0.048*	
H8B	−0.0292	1.3855	0.0537	0.048*	
C9	0.1259 (3)	1.41889 (18)	0.13900 (11)	0.0385 (5)	
H9	0.0747	1.4784	0.1752	0.046*	
C10	0.2186 (3)	1.30721 (18)	0.17856 (11)	0.0383 (4)	
H10A	0.1717	1.2869	0.2257	0.046*	
H10B	0.3256	1.3371	0.1863	0.046*	
C11	0.2141 (2)	1.17906 (16)	0.12901 (9)	0.0270 (3)	
H11	0.3111	1.1711	0.1008	0.032*	
C12	0.1830 (2)	1.04664 (17)	0.17179 (8)	0.0292 (4)	
C13	0.1586 (3)	0.79979 (17)	0.17246 (10)	0.0333 (4)	
H13A	0.1398	0.7249	0.1382	0.040*	
H13B	0.0663	0.8092	0.2029	0.040*	
C14	0.2970 (3)	0.76303 (19)	0.22022 (10)	0.0353 (4)	
C15	0.3801 (3)	0.6167 (3)	0.31610 (14)	0.0548 (6)	
H15A	0.4280	0.6929	0.3425	0.066*	
H15B	0.4602	0.5718	0.2869	0.066*	
C16	0.3116 (4)	0.5171 (3)	0.36799 (15)	0.0711 (9)	
H16A	0.3933	0.4765	0.3970	0.107*	
H16B	0.2580	0.4457	0.3414	0.107*	
H16C	0.2390	0.5641	0.3994	0.107*	

### Atomic displacement parameters (Å^2^)

**Table:**

	*U*^11^	*U*^22^	*U*^33^	*U*^12^	*U*^13^	*U*^23^
O1	0.0530 (10)	0.0443 (7)	0.0533 (8)	0.0063 (8)	−0.0280 (8)	0.0017 (7)
O2	0.0756 (12)	0.0458 (7)	0.0226 (6)	−0.0069 (8)	0.0034 (6)	0.0001 (5)
O3	0.0718 (13)	0.0293 (6)	0.0720 (11)	−0.0031 (8)	0.0311 (10)	0.0004 (7)
O4	0.0435 (9)	0.0418 (7)	0.0425 (7)	−0.0019 (7)	−0.0044 (6)	0.0179 (6)
O5	0.0443 (10)	0.0727 (12)	0.0861 (13)	−0.0162 (10)	−0.0148 (9)	0.0430 (10)
N1	0.0319 (9)	0.0271 (7)	0.0328 (7)	0.0039 (6)	−0.0047 (6)	0.0007 (5)
N2	0.0402 (10)	0.0256 (6)	0.0240 (6)	−0.0003 (6)	0.0007 (6)	0.0043 (5)
C1	0.0333 (10)	0.0243 (7)	0.0250 (7)	−0.0037 (7)	0.0014 (6)	−0.0006 (6)
C2	0.0468 (13)	0.0303 (8)	0.0387 (9)	0.0031 (9)	0.0036 (9)	−0.0004 (7)
C3	0.0591 (16)	0.0382 (9)	0.0462 (11)	0.0012 (10)	0.0159 (11)	−0.0116 (8)
C4	0.0744 (17)	0.0461 (10)	0.0279 (8)	−0.0115 (11)	0.0115 (10)	−0.0091 (8)
C5	0.0553 (14)	0.0376 (9)	0.0255 (8)	−0.0098 (9)	−0.0031 (8)	0.0026 (7)
C6	0.0348 (10)	0.0267 (7)	0.0250 (7)	−0.0057 (7)	−0.0024 (7)	0.0008 (6)
C7	0.0371 (11)	0.0287 (8)	0.0316 (8)	−0.0007 (7)	−0.0070 (7)	0.0061 (6)
C8	0.0391 (12)	0.0301 (8)	0.0516 (10)	0.0083 (8)	0.0007 (9)	0.0006 (8)
C9	0.0464 (13)	0.0264 (8)	0.0428 (10)	0.0008 (8)	0.0135 (9)	−0.0059 (7)
C10	0.0432 (12)	0.0319 (8)	0.0398 (9)	−0.0016 (8)	0.0003 (9)	−0.0115 (7)
C11	0.0292 (10)	0.0258 (7)	0.0259 (7)	−0.0001 (7)	−0.0024 (6)	−0.0019 (6)
C12	0.0322 (10)	0.0314 (7)	0.0239 (7)	−0.0010 (7)	−0.0028 (7)	0.0007 (6)
C13	0.0387 (11)	0.0300 (8)	0.0311 (8)	−0.0042 (7)	−0.0018 (7)	0.0077 (6)
C14	0.0365 (12)	0.0328 (8)	0.0366 (9)	−0.0016 (8)	−0.0015 (8)	0.0095 (7)
C15	0.0501 (15)	0.0564 (12)	0.0579 (13)	0.0070 (12)	−0.0091 (11)	0.0248 (11)
C16	0.080 (2)	0.0770 (17)	0.0566 (14)	0.0242 (17)	0.0073 (14)	0.0332 (13)

### Geometric parameters (Å, °)

**Table:**

O1—C7	1.238 (2)	C5—H5	0.9300
O2—C12	1.219 (2)	C6—C7	1.489 (3)
O3—C9	1.415 (2)	C8—C9	1.517 (3)
O3—H3	0.841 (10)	C8—H8A	0.9700
O4—C14	1.322 (2)	C8—H8B	0.9700
O4—C15	1.455 (3)	C9—C10	1.521 (3)
O5—C14	1.193 (3)	C9—H9	0.9800
N1—C7	1.336 (2)	C10—C11	1.534 (2)
N1—C11	1.471 (2)	C10—H10A	0.9700
N1—C8	1.471 (2)	C10—H10B	0.9700
N2—C12	1.359 (2)	C11—C12	1.522 (2)
N2—C1	1.430 (2)	C11—H11	0.9800
N2—C13	1.459 (2)	C13—C14	1.513 (3)
C1—C2	1.397 (3)	C13—H13A	0.9700
C1—C6	1.402 (2)	C13—H13B	0.9700
C2—C3	1.376 (3)	C15—C16	1.472 (3)
C2—H2	0.9300	C15—H15A	0.9700
C3—C4	1.381 (3)	C15—H15B	0.9700
C3—H3A	0.9300	C16—H16A	0.9600
C4—C5	1.377 (3)	C16—H16B	0.9600
C4—H4	0.9300	C16—H16C	0.9600
C5—C6	1.401 (2)		
			
C9—O3—H3	110 (2)	C8—C9—H9	109.1
C14—O4—C15	116.28 (18)	C10—C9—H9	109.1
C7—N1—C11	125.27 (15)	C9—C10—C11	106.17 (15)
C7—N1—C8	122.48 (16)	C9—C10—H10A	110.5
C11—N1—C8	111.83 (14)	C11—C10—H10A	110.5
C12—N2—C1	124.78 (13)	C9—C10—H10B	110.5
C12—N2—C13	116.22 (13)	C11—C10—H10B	110.5
C1—N2—C13	118.86 (13)	H10A—C10—H10B	108.7
C2—C1—C6	119.74 (15)	N1—C11—C12	108.85 (15)
C2—C1—N2	117.34 (16)	N1—C11—C10	103.58 (14)
C6—C1—N2	122.65 (16)	C12—C11—C10	112.28 (13)
C3—C2—C1	120.47 (19)	N1—C11—H11	110.6
C3—C2—H2	119.8	C12—C11—H11	110.6
C1—C2—H2	119.8	C10—C11—H11	110.6
C4—C3—C2	120.6 (2)	O2—C12—N2	120.99 (15)
C4—C3—H3A	119.7	O2—C12—C11	123.37 (15)
C2—C3—H3A	119.7	N2—C12—C11	115.62 (13)
C3—C4—C5	119.21 (18)	N2—C13—C14	112.56 (16)
C3—C4—H4	120.4	N2—C13—H13A	109.1
C5—C4—H4	120.4	C14—C13—H13A	109.1
C4—C5—C6	121.87 (19)	N2—C13—H13B	109.1
C4—C5—H5	119.1	C14—C13—H13B	109.1
C6—C5—H5	119.1	H13A—C13—H13B	107.8
C5—C6—C1	118.02 (17)	O5—C14—O4	125.18 (19)
C5—C6—C7	116.15 (16)	O5—C14—C13	124.73 (17)
C1—C6—C7	125.82 (14)	O4—C14—C13	110.08 (18)
O1—C7—N1	120.98 (18)	O4—C15—C16	108.4 (2)
O1—C7—C6	120.85 (16)	O4—C15—H15A	110.0
N1—C7—C6	118.12 (16)	C16—C15—H15A	110.0
N1—C8—C9	103.94 (16)	O4—C15—H15B	110.0
N1—C8—H8A	111.0	C16—C15—H15B	110.0
C9—C8—H8A	111.0	H15A—C15—H15B	108.4
N1—C8—H8B	111.0	C15—C16—H16A	109.5
C9—C8—H8B	111.0	C15—C16—H16B	109.5
H8A—C8—H8B	109.0	H16A—C16—H16B	109.5
O3—C9—C8	112.32 (18)	C15—C16—H16C	109.5
O3—C9—C10	113.62 (19)	H16A—C16—H16C	109.5
C8—C9—C10	103.30 (14)	H16B—C16—H16C	109.5
O3—C9—H9	109.1		
			
C12—N2—C1—C2	137.2 (2)	N1—C8—C9—O3	90.74 (19)
C13—N2—C1—C2	−38.4 (3)	N1—C8—C9—C10	−32.09 (19)
C12—N2—C1—C6	−48.8 (3)	O3—C9—C10—C11	−90.0 (2)
C13—N2—C1—C6	135.63 (19)	C8—C9—C10—C11	32.0 (2)
C6—C1—C2—C3	−2.3 (3)	C7—N1—C11—C12	−69.2 (2)
N2—C1—C2—C3	171.88 (19)	C8—N1—C11—C12	118.12 (16)
C1—C2—C3—C4	−0.4 (3)	C7—N1—C11—C10	171.13 (18)
C2—C3—C4—C5	1.9 (4)	C8—N1—C11—C10	−1.53 (19)
C3—C4—C5—C6	−0.8 (3)	C9—C10—C11—N1	−19.1 (2)
C4—C5—C6—C1	−1.8 (3)	C9—C10—C11—C12	−136.39 (17)
C4—C5—C6—C7	178.27 (19)	C1—N2—C12—O2	−178.8 (2)
C2—C1—C6—C5	3.3 (3)	C13—N2—C12—O2	−3.2 (3)
N2—C1—C6—C5	−170.55 (18)	C1—N2—C12—C11	2.2 (3)
C2—C1—C6—C7	−176.78 (18)	C13—N2—C12—C11	177.84 (17)
N2—C1—C6—C7	9.4 (3)	N1—C11—C12—O2	−111.0 (2)
C11—N1—C7—O1	−176.41 (17)	C10—C11—C12—O2	3.1 (3)
C8—N1—C7—O1	−4.5 (3)	N1—C11—C12—N2	68.0 (2)
C11—N1—C7—C6	1.1 (3)	C10—C11—C12—N2	−177.93 (18)
C8—N1—C7—C6	173.01 (16)	C12—N2—C13—C14	−69.9 (2)
C5—C6—C7—O1	32.0 (3)	C1—N2—C13—C14	106.03 (19)
C1—C6—C7—O1	−147.9 (2)	C15—O4—C14—O5	−2.1 (3)
C5—C6—C7—N1	−145.53 (19)	C15—O4—C14—C13	176.49 (19)
C1—C6—C7—N1	34.6 (3)	N2—C13—C14—O5	−17.2 (3)
C7—N1—C8—C9	−151.47 (18)	N2—C13—C14—O4	164.23 (16)
C11—N1—C8—C9	21.4 (2)	C14—O4—C15—C16	177.9 (2)

### Hydrogen-bond geometry (Å, °)

**Table:**

*D*—H···*A*	*D*—H	H···*A*	*D*···*A*	*D*—H···*A*
O3—H3···O1^i^	0.84 (1)	1.95 (1)	2.782 (2)	173 (4)

Symmetry codes: (i) *x*+1/2, −*y*+5/2, −*z*.
